# Association of cardiometabolic risk factors with insulin resistance in overweight and obese children

**DOI:** 10.1186/s12902-022-01245-7

**Published:** 2022-12-19

**Authors:** Elnaz Daneshzad, Sayeh Rostami, Fatemeh Aghamahdi, Armita Mahdavi-Gorabi, Mostafa Qorbani

**Affiliations:** 1 Non-Communicable Diseases Research Center, Alborz University of Medical Sciences, Karaj, Iran; 2 Student Research Committee, Alborz University of Medical Sciences, Karaj, Iran; 3 Department of Pediatric Endocrinology, School of Medicine, Alborz University of Medical Sciences, Karaj, Iran; 4 Probiotic Research Center, Alborz University of Medical Sciences, Karaj, Iran; 5 Endocrinology and Metabolism Research Center, Endocrinology and Metabolism Clinical Sciences Institute, Tehran University of Medical Sciences, Tehran, Iran

**Keywords:** Cardiometabolic risk factors, Insulin resistance, Overweight, Obesity, Child

## Abstract

**Introduction:**

Regarding the increased prevalence of obesity among children and adolescents, and the impact of obesity on insulin resistance (IR) and other metabolic disorders, this study was performed to determine the association of cardiometabolic risk factors (CMRFs) with IR in overweight and obese children.

**Method:**

In this cross-sectional study 150 overweight and obese children (BMI ≥ 85^th^ and BMI ≥ 95^th^ age-sex specific percentile) and adolescents were selected via convenient sampling method from Endocrinology clinic in Karaj; Iran in 2020. Anthropometric indices, lipid profile, fasting blood glucose (FBG), and Homeostatic Model Assessment for Insulin Resistance (HOMA-IR) were evaluated. IR was defined as HOMA-IR ≥ 2.6. Multivariable linear and logistic regression model was used to assess the association of CMRFs with insulin level and IR respectively.

**Results:**

The mean age of children was 10.37 (± 2.6) years. Fifty-four percent of the participants were girls. IR was increased through increasing age (*P* < 0.001). In the multivariate logistic regression model, by increasing each unit increment in waist circumference (OR: 1.03, 95% CI: 1.01–1.06), wrist circumference (OR: 1.47, 95% CI: 1.06–2.02) total cholesterol (OR: 1.01, 95% CI: 1.003–1.03) and FBG (OR: 1.11, 95% CI: 1.05–1.18) the odds of IR increased significantly. Moreover, in the adjusted linear regression model, HOMA-IR was associated significantly with waist to height ratio (β: 2.45), and FBG (β: 0.02).

**Conclusion:**

There was a significant association between some CMRFS with IR in overweight and obese children.

**Supplementary Information:**

The online version contains supplementary material available at 10.1186/s12902-022-01245-7.

## Introduction

There are forty-one million obese and overweight children under the age of 5 in the world which about 50% of them live in Asia. Also, there are more than 340 million overweight and obese and children above 5 years in the world [1]. In 2019, the prevalence of obesity among children was about 115 million children in the world [2]. Adult obesity is the biggest concern in these children because only a small number of them can lose weight and reach their ideal weight [3]. Weight gain has been increased in Iranian child's community so that about one fifth of children and adolescents with the age of 6 to 18 years old have abdominal obesity [4]. Obese children are prone to develop chronic diseases such as cardiovascular diseases, insulin resistance (IR), metabolic syndrome (MetS), and diabetes [2]. Insulin resistance occurs as a physiological and transient phenomenon during puberty which occurs through the anabolic effects of insulin and growth hormone [5]. Obesity and puberty are important factors for the development of IR, therefore both gender and pubertal status should be considered when evaluating IR in obese children [5]. Persistent weight gain and hyperinsulinemia makes children susceptible to some chronic disorders such as hypertension, polycystic ovaries syndrome, sleep apnea, non-alcoholic fatty liver, and depression in adulthood [6]. Fat accumulation in the adipocytes triggers oxidative stress and insulin resistance [7] which is one of the important cardiometabolic disorders markers in children, through the release of some inflammatory cytokines and decreasing level of the adiponectin [8]. Therefore, early diagnosis, prevention, and treatment of obesity, and insulin resistance can help to manage obesity-related complications. Although body mass index (BMI) has been used as a usual general obesity assessment index, it cannot determine the fat distribution. Also, at an early age, the contribution of lean body mass and fat mass varies according to race, gender, and maturity [9, 10]. Moreover, according to a previous studies, it seems that other anthropometric indices such as waist circumference (WC), neck circumference (NC), and waist-to-height ratio (WHtR) could present an additional useful method of cardiometabolic risk assessment [11, 12].

The present cross-sectional study was conducted to determine the association between insulin resistance (IR) and some anthropometric indices and cardiometabolic risk factors in 150 Iranian overweight and obese children.

## Method

### Study population

The present cross-sectional study was conducted among 150 overweight and obese children and adolescents who were referred to Endocrinology Clinic of Imam Ali Hospital; Karaj; Iran in 2020. The main reason of referral of the study population to the Endocrinology Clinic was assessment of weight status to recognize if children were normal, overweight or obese. Sample size was calculated according to correlation coefficient formula by considering alpha and beta error 0.05 and 0.2, respectively. According to a cross-sectional study among adolescent students and by considering correlation between WC and insulin level 0.23 sample size was determine 150 subjects [13].

Overweight and obese children (BMI ≥ 85% and BMI ≥ 95% of percentile according to age and gender, respectively) less than 18 years, who their parents have accepted to continue the study, were included. Patients who consumed corticosteroids, growth hormone, Metformin, Glibenclamide, or any medicine for treatment of obesity based on the medical history and also who had hypothyroidism or abnormal liver function tests were excluded from the study. The informed written consents were filled out by parents of the patients. The present study was approved by the ethical committee of Alborz University of Medical Sciences (IR.ABZUMS.REC.1399.198). Demographic information including age, gender, and family history of diseases was collected by demographic questionnaire.

### Anthropometric measurements

Weight was evaluated with the least clothes by a SECA scale with 0.5 kg of accuracy. Height was metered by a non-elastic tape measure with a 0.5 cm accuracy. Finally, BMI was calculated by weight; kg divided by the height; m^2^. Waist circumference was measured using a tape measure at the level of the umbilicus with 0.1 cm of accuracy and without clothes. The size of right wrist circumference was checked at the level of the distal prominence of radius and ulna bones with the accuracy of 0.1 cm. NC was assessed at the level of cricoid cartilage using a non-elastic tape measure with an accuracy of 0.1 cm. Waist to height ratio (WHtR) was calculated by waist circumference (cm) dividing height (cm).

### Biochemical markers measurement

Biochemical markers including insulin level, fasting blood glucose (FBG), triacylglycerol (TG), total cholesterol, high- and low-density lipoprotein cholesterols (HDL-C and LDL-C) were measured after 12-h fasting. All biochemical markers were measured using commercial enzymatic reagent (Pars Azmoon, Tehran, Iran). IR was determined by Homeostatic Model Assessment for Insulin Resistance; HOMA-IR (fasting glucose concentration; mg/dl × fasting insulin concentration; IU/L / 405). HOMA-IR ≥ 2.6 reveals IR [14].

### Statistical analysis

Data presented as mean (SD) for quantitative variables and number (%) for qualitative variables, which were obtained using T-test and chi-square test, respectively. According to skewed distribution, spearman correlation coefficient was determined for the correlation of anthropometric and biochemical assessments with insulin level and HOMA-IR. Linear regression analysis was conducted to find the association between anthropometric and biochemical measures and insulin level in obese children based on logarithmic variables. Multivariate logistic regression analysis was conducted to find the association between anthropometric and biochemical measures with IR in obese children in 2 models; crude model and adjusted model (adjusted for age and gender, moreover for BMI in biochemical markers). Statistical analysis of all data were done by SPSS statistical software (SPSS Inc., Chicago, IL, USA, version 24). *P* values less than 0.05 were considered statistically significant.

## Results

### Characterization of the study population

One hundred and fifty participants with the mean age of 10.37 ± 2.69 years were assessed, which the characteristics of them is illustrated in Table 1. Of these 150 students, 45 subjects were overweight, while 105 were obese. According to this table, subjects with more IR had more age and weight (*P* < 0.001), WHtR (*P* = 0.007) and BMI (*P* = 0.01) as well as wrist circumference (*P* = 0.002). Moreover, the levels of TG, cholesterol and FBG in children with IR were higher than those who had not involved with IR (*P* < 0.05).

**Table 1:** Characteristics of the participants

Variables	Total	Insulin Resistance		*P*-value
		**Yes *N* = 108**	**No *N* = 42**	
**Gender;** n (%)				
Female	81 (54.0)	56 (51.9)	25 (59.5)	0.39
Male	69 (46.0)	52 (48.1)	17 (40.5)	
**Age** (yr)	10.3 (2.6)	10.9 (2.5)	9.0 (2.4)	** < 0.001**
**Birth weight** (g)	3127.3 (712.4)	3194 (686.2)	2955 (757.1)	0.65
**Weight** (kg)	55.4 (19.2)	59.5 (19.3)	45.1 (14.5)	** < 0.001**
**BMI** (kg/m^2^)	26.0 (4.7)	26.8 (4.9)	23.8 (3.8)	**0.01**
**WC** (cm)	88.1 (53.2)	92.5 (61.0)	76.7 (12.0)	0.10
**NC** (cm)	33.4 (4.6)	39.9 (5)	32.2 (4.5)	0.058
**Wrist Circumference** (cm)	16.4 (2.5)	16.8 (2.7)	15.3 (1.3)	**0.002**
**WHtR**	0.59 (0.06)	0.60 (0.06)	0.57 (0.04)	**0.007**
**TG** (mg/dl)	134.9 (71.3)	143.6 (72.0)	112.4 (65)	**0.016**
**Cholesterol** (mg/dl)	162.8 (33.8)	170.1 (33.4)	154.6 (32.7)	**0.011**
**LDL** (mg/dl)	96.3 (63.1)	100.3 (72.4)	86 (24.3)	0.211
**HDL** (mg/dl)	47.4 (13.0)	47.1 (13.1)	48.2 (12.7)	0.648
**FBG** (mg/dl)	90.2 (9.6)	92.1 (9.9)	85.2 (6.7)	** < 0.001**
**Insulin** (μIU/mL)	18.5 (13.5)	21.4 (11.1)	11.2 (16.3)	** < 0.001**
**HOMA-IR**	4.1 (2.87)	5 (2.8)	1.7 (0.6)	** < 0.001**

Data presented as mean (SD) for quantitative variables and number (%) for qualitative variables, which were obtained using T-test and chi-square test, respectively

*BMI* Body Mass Index, *WC* Waist Circumference, *WHtR* waist-to-height ratio, *NC* Neck Circumference, *TG* Triacylglycerols, *LDL* Low-density Lipoprotein, *HDL* High-density Lipoprotein, *FBG* Fasting Blood Glucose, *HOMA-IR* Homeostatic Model Assessment for Insulin Resistance

### Correlation of biochemical characteristics with IR

Spearman correlation coefficient for the correlation of insulin level and HOMA-IR with the anthropometric and biochemical measure is indicated in Table 2. There was a positive significant correlation between WHtR and insulin level, as well as HOMA-IR (*P* < 0.001). A similar correlation was seen between insulin level and zBMI, WC, NC, and wrist circumference (*P* < 0.05), and also, between HOMA-IR and zBMI, WC, NC, and wrist circumference (*P* < 0.05). Furthermore, a significant correlation was found between insulin level and TG, HDL-C, and FBG (*P* < 0.05). However, HOMA-IR had a same correlation only with and TG and FBG (*P* < 0.05).

**Table 2:** Correlation between quantitative variables

Variables					
**Anthropometric indices**					
	**zBMI**	**WC**	**WHtR**	**NC**	**Wrist Circumference**
**Insulin level**	0.445^*^ (0.301 – 0.579)	0.459^*^ (0.321 – 0.586)	0.353^*^ (0.195 – 0.512)	0.368^*^ (0.212 – 0.511)	0.378^*^ (0.229 – 0.501)
**HOMA-IR**	0.403^*^ (0.248 – 0.541)	0.416^*^ (0.242 – 0.559)	0.307^*^ (0.156 – 0.449)	0.316^*^ (0.158 – 0.479)	0.379^*^ (0.217 – 0.513)
**Biochemical assessment**					
	**TG**	**Cholesterol**	**LDL**	**HDL**	**FBG**
**Insulin level**	0.239^*^ (0.072 – 0.384)	0.116 (-0.043 – 0.260)	0.077 (-0.086 – 0.250)	-0.217^*^ (-0.365 – -0.054)	0.332^*^ (0.174 – 0.473)
**HOMA-IR**	0.242^*^ (0.066 – 0.392)	0.142 (-0.021 – 0.282)	0.084 (-0.073 – 0.234)	-0.149 (-0.297 – 0.018)	0.446^*^ (0.307 – 0.579)

*zBMI* Z-score of Body Mass Index, *WHtR* Waist to height ratio, *NC* Neck Circumference, *HOMA-IR* Homeostatic Model Assessment for Insulin Resistance, *TG* Triacylglycerols, *LDL* Low-density Lipoprotein, *HDL* High-density Lipoprotein, *FBG* Fasting Blood Glucose

^*^ *P* value < 0.05. *P* values obtained by Spearman Correlation. The numbers in parenthesis show the 95% confidence intervals

### Association of cardiometabolic risk factors with IR

Table 3 presents the association between anthropometric and biochemical measures with insulin level and HOMA-IR in obese children using linear regression analysis. Based on the results, there was a positive significant association between insulin level and Z score of BMI (zBMI), NC, TG, FBG and WHtR even after adjusting cofounders (*P* = 0.001, *P* = 0.039, *P* = 0.038, *P* = 0.028 and *P* < 0.001, respectively). There was a positive significant association between insulin level and wrist circumferences (*P* < 0.001), and an inverse association with HDL (*P* = 0.034). However, these significant associations were disappeared after adjusting cofounders. Moreover, there was a positive significant association between HOMA-IR and zBMI (*P* = 0.001), WHtR (*P* = 0.001), wrist circumferences (*P* = 0.005), TG (*P* = 0.014), and FBG (*P* < 0.001) even after adjusting cofounders.

**Table 3:** Association between anthropometric and biochemical measures with insulin level and HOMA-IR in obese children: linear regression analysis

**Variables**	**Ln Insulin level**					**Ln HOMA-IR**				
	**β**	**95% CI**	***P*-value**	**R2**	**Adjusted R2**	**β**	**95% CI**	***P*-value**	**R2**	**Adjusted R2**
**zBMI**										
Crude model	0.275	(0.190, 0.360)	** < 0.001**	0.215	0.210	0.282	(0.186, 0.378)	** < 0.001**	0.185	0.180
Adjusted model	0.177	(0.077, 0.276)	**0.001**	0.279	0.265	0.188	(0.076, 0.301)	**0.001**	0.242	0.226
**WC** (cm)										
Crude model	0.002	(0.000, 0.004)	0.027	0.033	0.026	0.002	(0.000, 0.004)	**0.041**	0.028	0.021
Adjusted model	0.001	(-0.001, 0.003)	0.212	0.227	0.211	0.001	(-0.001, 0.003)	0.268	0.192	0.175
**WHtR**										
Crude model	3.038	(1.610, 4.467)	** < 0.001**	0.107	0.101	2.711	(1.097, 4.324)	**0.001**	0.069	0.063
Adjusted model	2.781	(1.513, 4.050)	** < 0.001**	0.307	0.293	2.453	(0.984, 3.923)	**0.001**	0.242	0.226
**NC** (cm)										
Crude model	0.036	(0.017, 0.054)	** < 0.001**	0.090	0.084	0.032	(0.011, 0.053)	**0.003**	0.059	0.053
Adjusted model	0.019	(0.001, 0.038)	**0.039**	0.241	0.225	0.016	(-0.005, 0.037)	0.137	0.197	0.181
**Wrist circumference** (cm)										
Crude model	0.071	(0.035, 0.108)	** < 0.001**	0.092	0.086	0.082	(0.042, 0.123)	** < 0.001**	0.099	0.093
Adjusted model	0.001	(-0.001, 0.003)	0.212	0.227	0.211	0.058	(0.018, 0.098)	**0.005**	0.228	0.212
**TG** (mg/dl)										
Crude model	0.002	(0.001, 0.004)	** < 0.001**	0.081	0.075	0.003	(0.001, 0.004)	** < 0.001**	0.091	0.084
Adjusted model	0.001	(0.000, 0.002)	**0.038**	0.301	0.281	0.002	(0.000, 0.003)	**0.014**	0.273	0.253
**Cholesterol** (mg/dl)										
Crude model	0.002	(-0.001, 0.004)	0.264	0.008	0.002	0.003	(0.000, 0.006)	0.096	0.019	0.012
Adjusted model	0.001	(-0.002, 0.003)	0.488	0.282	0.262	0.002	(-0.001, 0.005)	0.146	0.253	0.232
**LDL** (mg/dl)										
Crude model	0.000	(-0.001, 0.002)	0.797	0.000	-0.006	0.001	(-0.001, 0.002)	0.643	0.001	-0.005
Adjusted model	0.000	(-0.001, 0.001)	0.795	0.280	0.260	0.001	(-0.001, 0.002)	0.980	0.242	0.221
**HDL** (mg/dl)										
Crude model	-0.008	(-0.015, -0.001)	**0.034**	0.030	0.024	-0.004	(-0.013, 0.004)	0.290	0.008	0.001
Adjusted model	-0.004	(-0.011, 0.002)	0.215	0.287	0.267	0.000	(-0.008, 0.007)	0.900	0.242	0.221
**FBG** (mg/dl)										
Crude model	0.017	(0.007, 0.026)	**0.001**	0.073	0.067	0.026	(0.016, 0.036)	** < 0.001**	0.149	0.144
Adjusted model	0.010	(0.001, 0.018)	**0.028**	0.303	0.284	0.020	(0.010, 0.029)	** < 0.001**	0.321	0.302

*CI* confidence interval, *HOMA-IR* Homeostatic Model Assessment for Insulin Resistance, *zBMI* Z score of body mass index, *WC* waist circumference, *WHtR* Waist to height ratio, *NC* neck circumference, *TG* Triacylglycerols, *LDL* low-density lipoprotein, *HDL* high-density lipoprotein, *FBG* fasting blood glucose

Adjusted model: Variables were controlled for age and gender. Moreover, biochemical variables were adjusted for one more confounder (BMI)

*P*-values obtained by linear regression analysis. Hence the distribution of insulin level and HOMA-IR were not normal, the analyses were performed using by logarithmic variables (Ln)

Table 4 indicates the association between anthropometric and biochemical measures with IR in obese children using logistic regression analysis. There were a positive significant association between WC (OR: 1.035; 95%CI: 1.007, 1.063), wrist circumference (OR: 1.470; 95%CI: 1.067, 2.024), FBG (OR: 1.115; 95%CI: 1.048, 1.187) and cholesterol (OR: 1.016; 95%CI: 1.003, 1.029), and IR (*P* < 0.05).

**Table 4:** Association between anthropometric and biochemical measures with insulin resistance in obese children: logistic regression analysis

Variables	OR	95% Confidence Interval	*P* value
**zBMI**			
Crude model	2.184	1.371; 3.479	**0.001**
Adjusted model	1.557	0.922; 2.629	0.098
**WC** (cm)			
Crude model	1.047	1.017; 1.078	**0.021**
Adjusted model	1.035	1.007; 1.063	**0.013**
**NC** (cm)			
Crude model	1.099	0.998; 1.209	0.054
Adjusted model	1.027	0.941; 1.121	0.554
**Wrist circumference** (cm)			
Crude model	1.660	1.264; 2.180	** < 0.001**
Adjusted model	1.470	1.067; 2.024	**0.018**
**TG** (mg/dl)			
Crude model	1.007	1.001; 1.014	**0.019**
Adjusted model	1.005	0.998; 1.011	0.177
**Cholesterol** (mg/dl)			
Crude model	1.015	1.003; 1.027	**0.015**
Adjusted model	1.016	1.003; 1.029	**0.018**
**LDL** (mg/dl)			
Crude model	1.010	0.997; 1.023	0.125
Adjusted model	1.011	0.996; 1.026	0.141
**HDL** (mg/dl)			
Crude model	0.994	0.967; 1.021	0.646
Adjusted model	1.002	0.972; 1.033	0.888
**FBG** (mg/dl)			
Crude model	1.130	1.063; 1.200	** < 0.001**
Adjusted model	1.115	1.048; 1.187	**0.001**

*zBMI* Z score of body mass index, *WC* waist circumference, *NC* neck circumference, *TG* Triacylglycerols, *LDL* low-density lipoprotein, *HDL* high-density lipoprotein, *FBG* fasting blood glucose

Adjusted model: Variables were controlled for age and gender. Moreover, biochemical variables were adjusted for one more confounder (BMI)

Insulin resistance (IR) was determined by HOMA-IR ≥ 2.6 (Homeostatic Model Assessment for Insulin Resistance)

*P*-values obtained by logistic regression analysis

## Discussion

The present study was conducted among overweight and obese children to find the association between IR and several cardiometabolic risk factors. Based on the results, there was a significant correlation between anthropometric indices including zBMI, WHtR, WC, NC, and wrist circumference with insulin level and HOMA-IR. There was a significant positive association between several anthropometric indices including zBMI, and WHtR with insulin level and HOMA-IR. Moreover, there was a significant positive association between WC, wrist circumference, total cholesterol, and FBG and IR among overweight and obese children and adolescents.

Hyperinsulinemia and insulin resistance play an essential role in development of cardiometabolic disorders such as obesity, diabetes mellitus an also MetS [15], according to previous study, children with metabolic syndrome had more WC than children without it [16], a finding that our results were consistent with it. The present study has shown that children with more HOMA-IR and insulin level had more ratio of waist to height. Muhammad Asif et al. revealed that WHtR was the best anthropometric index between various studied indices among 5946 Pakistani children with the age of 5–12 years old, regarding its independency to age and gender [17]. A study among 110 Mexican obese adolescents with the age of 8–16 years old has indicated that participants with MetS had more WHtR. The reported WHtR cut-off for insulin resistance diagnosis was 0.61 which is in line with our study (mean WHtR among participants with IR = 0.6) [18]. These findings may show the practical usefulness of WHtR for MetS diagnosis and hyperinsulineamia which suggested to evaluate in future studies.

Moreover, in line with our study, Capizzi et al. indicated that there was a significant association between wrist circumference and hyperinsulinemia among 477 overweight and obese children [19]. Also, a study among 72 women (mean age of 24.33 y) with polycystic ovary syndrome (PCOS) has shown that wrist circumference was significantly correlated with HOMA-IR [20]. Notably, in the mentioned study non-dominant wrist circumference was determined as the best anthropometrical marker which was correlated to HOMA-IR.

In fact, being overweight and excess weight gain induces numerous metabolic changes such as insulin resistance and elevated blood glucose [21]. Increased blood glucose leads to producing more insulin by the pancreas and finally causes hyperinsulinemia and IR which can develop chronic diseases such as cancer, diabetes, and cardiovascular diseases [22]. Obesity leads to hyperinsulinemia and IR through several physiological mechanisms such as hormone imbalance, deregulation of glucose and lipids metabolism, and inflammation [23, 24]. Based on our findings, there was a significant association between FBG and total cholesterol, and IR.

Disorders of insulin release or secretion lead to increasing the release of inflammatory factors such as interleukin-6 (IL-6) and C—reactive protein (CRP) which themselves may lead to increasing FBS, TG, and LDL-C [25, 26]. A study has presented that children with an FBS level of 86 to 99 mg/dl are significantly at the risk of insulin resistance and diabetes during childhood, even after controlling for cardiometabolic factors [27]. It seems even higher normal FBS may increase the risk of diabetes probably by increasing hepatic insulin resistance and early insulin response disorder [28]. As TG and HDL-C are two important lipid factors in the diagnosis of metabolic syndrome, a study among 9–16 years old overweight and obese children found that TG to HDL-C ratio was significantly correlated with HOMA-IR especially among children with HOMA-IR of more than 4 (*P* < 0.05) [29]. Also, TG level directly was correlated with IR in adults with normal glucose tolerance and inversely was associated with beta-cell function in individuals with dyslipidemia [30]. Also, a case–control study among 169 patients with non-alcoholic fatty liver disease (NAFLD) revealed that adherence to a diet with high food insulin index was associated with higher levels of FBS, TG, and LDL-C [31]. This issue shows the importance of dietary assessment in future studies.

The increase in the rate of obesity among children in the last few decades indicates more attention to IR and its related health outcomes such as developing type 2 diabetes in children. Insulin receptor mutations as a genetic factor may lead to IR. These mutations can induce the formation of abnormal glucose transporter 4 molecules or stimulate autoantibody production against insulin receptors [32]. Also, mutations in the lipid pathway have a significant role in the development of IR These mutations occurs in the peroxisomal proliferator-activated receptors (α, γ, δ), mutations in the adipocyte-derived hormones and their receptors such as leptin or adiponectin, mutation in the lipoprotein lipase gene, and other genes which are related with adipose tissue formation [33]. IR is characterized by the presence of one or more of the following items: impaired glucose tolerance, hyperglycemia, hyperinsulinemia, central obesity, hypertriglyceridemia, hypercholesterolemia and etc. [34]. IR is rising due to changing the lifestyle with lack of physical activities and sedentary life, adherence to high-calorie diets and western dietary patterns, and finally increasing obesity among children and adolescents [35]. The practical usefulness of the present study and similar ones is to identify hyperinsulinemic individuals and the related complications and finally to improve health status and decrease health outcome burden. The association of cardiometabolic risk factors with IR and hyperinsulinemia in overweight and obese adults is well studied but not in children and adolescents. Besides, the habits and lifestyles of childhood and adolescence often affect adulthood. Hence the importance of diagnosing future problems at an early age and based on simple and validated methods is clear. Anthropometric indices may be a useful substitute for clinical diagnosis of children who are at risk of heyperinsulinemia. Therefore, theses indices can be used instead of running expensive biochemical assessments. Also, they may play a role as diagnosis guidelines in clinics to improve lifestyle of children who are at high risk of cardiometabolic diseases, and can decrease health and economic costs. This is the first study that has been conducted among children and adolescents to find the association between IR and cardiometabolic risk factors. A systematic review of 13 articles revealed that the prevalence of hyperinsulinemia is higher among females especially in 7 of them [36]. This result is in line with the present study which showed the higher prevalence of hyperinsulinemia in girls. Hence, we controlled gender for main analysis to justify the gender effect on the outcome. However, several limitations should be considered in the present study. First, the sample size was small. Second, physical activity and dietary intake were not evaluated among children. Moreover, the levels of stress and mental health among children and adolescents is another factor that may contribute to BMI and metabolic changes [37]. Finally, the problem of using HOMA-2IR as a proxy to measure IR is that the insulin measurements trend to have high variability among the same subjects and populations. Therefore, using a specific cut-off value could lead to controversial findings.

## Conclusion

It has been shown that insulin level among children and adolcents was correlated with higher zBMI, WC, WHtR, NC, and wrist circumference, as well as more TG, and FBG. There was significant association between IR and WC, wrist circumference, cholesterol, and FBG, even after adjusting cinfiunder variables. It is suggested to conduct more well-designed surveys with resolving the mentioned limitations to find the real association between cardiometabolic risk factors and hyperinsulinemia/IR.

## Supplementary Information

**Additional file 1.** 

## Data Availability

The data that support the findings of this study are not publicly available due to restrictions and the privacy of research participants. However, it will be available on a reasonable request from the corresponding author.
